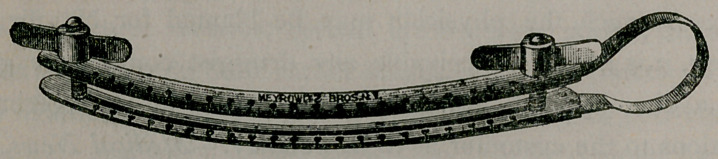# An Anatomical Bill

**Published:** 1886-12

**Authors:** 


### AN ANATOMICAL BILL.

The following bill, introduced by Hon. C. M. Candler, of DeKalb, and referred to the General Judiciary Committee, has been returned to the House by them with recommendation that it pass. It is hoped that the Legislature will see the importance of passing the bill:

"A BILL to be entitled An Act for the protection of cemeteries and burying places in this State, and to prevent and punish the unauthorized uses of and traffic in dead human bodies, and for the promotion of medical science by the distribution and use of unclaimed dead human bodies for scientific purposes through a Board created for that purpose, and for other purposes.

SECTION I. The General Assembly of the State of Georgia do hereby enact:

That the professors of anatomy, the demonstrators of anatomy and the deans of medical and dental schools and colleges of this State, which are now or may hereafter become incorporated under the laws of this State, shall be, and are hereby constituted a Board for the distribution and delivery of dead human bodies, hereinafter described, to and among such persons as under the provisions of this Act are entitled thereto.

The Professor of Anatomy in the medical department of the University of Georgia, at Augusta, shall call a meeting of said Board for organization, at Atlanta, at a time to be appointed by him, within thirty days after the approval of this act. The said Board shall have full power to establish rules and regulations for its government, and to appoint and remove its officers, and shall keep full and complete minutes of its transactions, and records shall also be kept under its direction of all bodies received and distributed by said Board, and of the persons or institutions to whom the same may be distributed, which minutes and records shall be open at all times to the inspection of each member of said Board, and of any Solicitor General or Solicitor of any city or county court in this State.

SEC. 2. That all public officers of this State and their assistants, and all officers and their deputies, of every county, city, town or other municipality, and of any and every prison, chain-gang, morgue, public hospital, penitentiary company in this State having charge or control over any dead human body or bodies *required to be buried at public expense*, are hereby required to notify the said Board of distribution, or such person or persons as may from time to time be designated in writing by said Board, or its duly authorized officer, whenever any such body or bodies come into his or their possession, charge or control, and shall, without fee or reward, deliver such body or bodies, or permit and suffer the said Board and its duly authorized agents, who may comply with the provisions of this Act, tc take and remove all such bodies, *to be used only within this State, solely for the advancement of medical science.*

PROVIDED, *that no such notice shall be given, nor shall any such body or bodies be delivered, if any person claiming to be and satisfying the authorities in charge of said body or bodies, that he or she is of any degree of kin, or is related by marriage to, or socially or otherwise connected with and interested in the deceased, shall claim the said body or bodies for burial, but it or they shall be at once surrendered for interment to such person, or shall be buried at public expense at the request of such claimant, if a relative by blood or a connection by marriage, provided he or she is financially unable to supply such body or bodies with burial.*

AND PROVIDED FURTHER, *that such notice shall not be given or such body be delivered if the deceased person was a traveler who died suddenly, in which case the said boay shall be properly buried.*

AND PROVIDED FURTHER, *that such body or bodies shall in each and every instance be held and kept by the person or persons having charge or control of it or them at least twenty-four hours after death, before delivery to said Board or its agent or agents, during which period notice of the death of such person or persons shall be posted at the court-house door of the county in which said body or bodies are so held.*

SEC. 3. That the said Board, or their duly authorized agent, may take and receive such bodies so delivered as aforesaid, and shall, upon receiving them, distribute and deliver them to and among the aforesaid schools or colleges for lectures and demonstrations by the said schools or colleges, the number assigned to each to be based upon the number of *bona fide* students in each dissecting or operative surgery class, which number shall be reported, under oath, by said schools or colleges to the Board at such times as it may direct.

PROVIDED, *that said schools or colleges, upon receiving them, and before any use is made of them, and without any dissection or*

*unnecessary mutilation thereof, shall cause them to be properly embalmed and decently kept and preserved for the period of sixty days after their reception, and shall deliver them properly prepared for burial to any person mentioned and described in section 2 of this Act, who shall claim such body within or before the expiration of said period of sixty days, and satisfy the authorities of said schools or colleges that he or she is such person as is entitled to said body under said section* 2.

If, at the expiration of said period of sixty days, said bodies have not been claimed for burial, in the manner and by the person or persons herein described, said bodies shall then be used for the purposes specified in this Act by said schools or colleges;

*And provided further*, that all of said bodies which have been so used and are no longer needed for the objects herein mentioned shall be decently interred by the said schools or colleges.

SEC. 4. The said Board may employ a carrier or carriers for the conveyance of said bodies, which shall be well enclosed within a suitable encasement, and carefully deposited free from public observation. Said carrier or carriers shall obtain receipts by name, or if the person be unknown by a description, for each body delivered by him, and shall deposit said receipts with the Secretary of said Board, who shall record and preserve the same.

SEC. 5. No school or college shall be allowed or permitted to receive any such body or bodies until a bond shall have been given to the Governor of this State, and his successors in office, by or in behalf of such school or college by its authorized officers, to be approved of by the Clerk of the Superior Court of the county in which said school or college may be situated, and to be filed in the office of said Clerk; which bond shall be in the sum of $5,000, conditioned that said body or bodies, which the said school or college shall receive thereafter, shall be used only in the manner herein described and only for the promotion of medical science within this State. Suits thereon shall be brought by the Solicitor-General of the circuit, in the name of the Governor; the recovery thereon to be used as a part of the educational fund of the State.

SEC. 6. That whosoever shall sell or buy such body or bodies, or any other dead human body, or in any way traffic in the same, or shall transmit or convey, or procure or cause to be procured to be transmitted or conveyed such body or bodies, or any other dead human body to any place outside of this State for purposes of sale or dissection, shall be guilty of a felony, and shall on conviction thereof be punished by imprisonment and hard labor in the penitentiary of this State not less than one nor more than ten years.

SEC. 7. That whosoever shall remove the dead body of a human being from any grave, or other place of interment, or from any vault, tomb, sepulchre, or from any other place, for the purpose of selling or dissecting the same, or from mere wantonness, shall be guilty of a felony, and on conviction thereof shall be punished by imprisonment and hard labor in the penitentiary not less than one year nor more than ten years, and any person who shall receive or purchase any dead human body, knowing it to have been so disinterred or removed from any tomb, vault or sepulchre, or such other place, for the purpose aforesaid, shall on conviction thereof receive the same punishment.

SEC. 8. Neither the State, nor any county or municipality, nor any officer, agent or servant thereof, shall be at any expense by reason of delivery or distribution of any such body or bodies, but all the expenses thereof shall be paid by those receiving the body or bodies, in such manner as may be specified or fixed by said Board.

SEC. 9. That any person having duties enjoined upon him by the provisions of this Act, who shall neglect, refuse or omit to perform the same as hereby required, shall be guilty of a misdemeanor, and on conviction thereof shall be punishedas prescribed in section 4705 of the Code of this State.

SEC. 10. That all laws and parts of laws in conflict with this Act be, and the same are hereby repealed."

---

### ITEMS.

THERE are more medical students in Atlanta now than we have ever known before.

DR. J. A. DUNWODY, of Brunswick, and Dr. W. C. Ashley, of Irwinville, were in Atlanta a few days since.

DR. ROBERT BATTEY, of Rome, will deliver an address before the Atlanta Society of Medicine on December 3d.

WE had a pleasant call from Dr. Charles A. Brooks, of Americus, a few days since. Dr. Brooks is one of the rising young men in Georgia.

THE ARCHIVES OF GYNÆCOLOGY, OBSTETRICS AND PÆDIATRICS, heretofore published bi-monthly, will hereafter be published as a monthly. The first number of the new series will appear in January next.

THE New York Academy of Medicine has received gifts and legacies amounting to nearly $200,000, the latest gift being $25,000 from Mrs. Woerishoffer. The Academy will soon begin the erection of a suitable building, as well as make other needed additions to its equipment. Mrs. Woerishoffer was led to make her gift to the Academy in consideration of the fact that by excluding politics and ethical strifes from its constitution and gatherings the Academy deserves the name of a purely and exclusively scientific body, and thereby the confidence of the public. —*Maryland Medical Journal.*

PROFESSIONAL SECRECY AND GOVERNMENT OFFICIALS.—The latest attempt to coerce physicians into a violation of professional secrecy has been undertaken by an official of the pension bureau, who, assuming (whether correctly or not is not to the point) that employees were getting to absent themselves for insufficient reasons, recently returned a certificate of disability signed by Dr. A. Y. P. Garnett, of Washington, with a demand for explicit information—meaning, of course, a statement of the nature of the case. Dr. Garnett very properly declined to furnish this information, on the ground that it would involve a violation of his professional obligations, and it is exceedingly gratifying to learn that he is sustained in this position by Secretary Lamar.—*New York Medical Journal.*

AN ANODYNE FOR USE IN VESICAL IRRITATION.—Dr. W. P. Copeland, of Eufaula, Ala., writes to the *Medical Record:* "In almost every community there are old men who suffer from enlarged prostates, accompanied with a chronic inflammation of the neck of the bladder, rendering them miserable sufferers and a care and anxiety to their friends and families. Having had the professional care of several of this class of cases, and dreading the tendency they so frequently acquire to the administration of opium for the relief of pain, I resorted to various washes for injecting the bladder, resulting in my adopting a solution of benzoate of soda, ten grains to one ounce of water, with twenty to thirty drops of the green tincture of gelseminum; this is warmed and injected by the patient through a soft rubber catheter, whenever the pain is severe, and the catheter withdrawn, leaving the medicine to be voided in twenty to thirty minutes; or, where he is not able to pass anything from the bladder, the catheter is re-introduced and the medicine allowed to escape. My experience with this treatment has been so satisfactory that I cannot refrain from giving it publicity to the profession."

PROBABLE SEPTIC ORIGIN OF PNEUMONIA.—A coincidence showing a probable septic origin of pneumonia is reported in the *Lancet*. On the 18th of October, a man and his wife were admitted into St. Thomas' Hospital, suffering from acute pneumonia of respectively three and four days' duration. Each was aged thirty-two years. The disease ran an acute course, being little influenced by treatment, and they died at the end of four days within a few hours of each other. At the post-mortem examinations, which were made on the same day, acute inflammation of the right lung were found in each; this had attacked chiefly the base in the case of the man and the apex in the woman. It would appear that they had left their house and moved into lodgings only two or three days before the commencement of the disease, on account of the bad smells, making it probable that the disease was of septic origin.—*Boston Medical and Surgical Journal.*

AN INTERESTING AND REMARKABLE ACCIDENT.—There came, accidentally, into our possession, a short time ago, a memorandum, signed by the late Joseph Pancoast, relating to a remarkable injury received by the late James P. White, the well-known gynecologist, of Buffalo. The note was as follows: "A front segment of the atlas vertebra, a little more than an inch on the superior margin, a little less below, with the facette which received the odontoid process. It was in the possession of Professor Granville S. Pattison, to whom it was loaned by Dr. White, to show Professors Joseph Pancoast and McClellan. It is probable that the traverse ligament retained its hold on the two extremities of the remaining fragment of the atlas, thus protecting the spinal marrow from injury. This bone in possession of Professor Pattison I repeatedly saw and carefully examined; he exhibited it to his class, and it was mislaid or lost. At the request of Professor White, I make this statement of facts. This bone was in our possession in 1838–39–40, or thereabout. I then understood and believed (since confirmed by conversation with Professor White) that it came from his throat, coming out through the mouth as a consequence of ulceration, the result of an accident while riding in a stage-coach on the morning of December 17, 1837. The bone was discharged at the expiration of forty-five days after receipt of the injury." This statement, signed "Joseph Pancoast," records an interesting and remarkable accident which, so far as we know, has not been made public, and of which there are only one or two instances mentioned in literature.—*Medical News, Nov. 27th, 1886.*

INTRA-UTERINE GALVANO-CAUTERY.—We have received from Dr. G. Apostoli, of Paris, some preliminary notes giving the results of his observations upon the use of the intra-uterine galvano-cautery in the treatment of certain uterine affections, of which the following are the chief: Uterine fibromata, certain forms of chronic metritis, certain intra-uterine polypi, uniloular cysts of the ovary in their earlier stages, chronic cellulitis of the broad ligament, sub-acute and chronic peri-metritis, peri-uterine hæmatocele and extra-uterine pregnancy. It is claimed for this mode of treatment that it is scientifically exact, since the strength of the current may be accurately measured in milliamperes; that it is painless, since the covering of potter's earth, which the author recommends, for the external electrode prevents the excessive pain which would otherwise accompany its application; that it is harmless, as proven by the author's experience with 200 cases; and more than all, that it is curative, since, in 95 per cent. of all cases in which it was employed, satisfactory results followed. The time occupied by the treatment varies from three to nine months. The apparatus necessary for this method of procedure consists of a galvanometer graduated up to 200 milliamperes, a battery capable of giving a current of from 100 to 200 milliamperes in intensity, an intra-uterine electrode of platinum insulated by a celluloid covering in its vaginal portion, an external electrode covered by potter's earth to prevent pain, burning and sloughing, strong, flexible cords which will not be liable to interrupt the current by breaking. The *technique* of the method involves many minute details, all of which, according to the author, are essential to its success.

AN UNUSUAL CASE OF POISONING.—The papers have recently related a fatal case of poisoning from an over-dose of podophyllum. A physician in Maine ordered in a prescription "Podophyllum, gr. ss." The last two letters were mistaken by the druggist for figures, and read "88." He thereupon dispensed the latter quantity, which was taken at one dose (as was presumably ordered for the correct quantity), death occurring soon after. The clinical history of the case is not given, but the toxic symptoms were probably those of an acute irritant poison. However much the physician may be blamed for his illegible writing, it is hardly conceivable any druggist could have given such a quantity of a powerful drug without at least verbal instructions to the customer as to its potency.—*Medical News.*

THE SO-CALLED COCAINE HABIT.—Apprehensions which some have entertained, lest there may develop a new and pernicious "habit," will be somewhat allayed by the discussion on the cocaine habit, so-called, which was held at the Neurological Society recently. For over a year the daily papers have been giving currency at times to shocking stories regarding victims of the cocaine habit, and a few reports of a similar kind have circulated among the profession. In Germany, especially, the subject has been studied by Erlenmeyer, and his contribution was supplemented by one of Dr. Borneman, and by that of Dr. Smidt, the latter having been read at the German Congress of Physicians and Naturalists. Altogether, therefore, the question of a cocaine habit has received very considerable ventilation. So far as any conclusions can now be drawn, they are to the effect that the cocaine habit is extremely rare, if it ever exists. The use of cocaine and morphine together, however, has been observed often, and a morbid habit, which has been termed "morphine-cocainism," has been developed. This habit is much more serious, physically and mentally, than the morphine habit alone. The addition of the cocaine seems especially to produce hallucinations and other alarming psychical troubles. German and American writers agree upon the baneful effects of this cocaine-morphine combination. The use of cocaine in helping patients to rid themselves of the opium habit is one, therefore, that should be employed with great caution.—*Medical Record, November 20, 1886.*

A NEW SCROTAL CLAMP.—The accompanying cut illustrates a new scrotal clamp devised by Dr. Ferdinand King, of this city, which is being used by some of our surgeons. He thus describes it in the *Medical Record* of November 13th:

"All the scrotal clamps heretofore invented have only served as *guides* to the surgeon, and their removal after cutting is always followed by profuse hemorrhage. With my clamp the operation is completed before its removal, and there is absolutely no hemorrhage. Another objection to other clamps has been the 'roughened' or 'serrated' inside surface to prevent slipping, which produces more or less laceration or contusion of the parts. To prevent slipping, on the inside of blades of my clamp are longitudinal grooves which receive a fold of scrotum, thus preventing any slipping whatever, and no bruising of the parts is to be encountered. I find that the cut edges, when the neighboring tissues are not bruised, always heal by first intention. Ordinarily I employ a woven-silk ligature, and apply simple carbolic dressings, but recently I have had splendid success by the antiseptic method. When the latter is employed I use the juniperized animal ligature, wash parts with 1,000 bichloride mercury solution, dress wound with iodoform cotton and gauze, held in place by figure-of-eight bandage. This dressing I allow to remain in position eight days. On its removal the wound is found to have healed by first intention and with little or no suppuration. In offering my clamp to the profession, I feel confident that its use will soon place excision of the scrotum as a radical cure for varicocele in the category of minor surgery."

PAINLESS PRODUCTION OF LOCAL ANÆSTHESIA.—Dr. J. Leonard Corning, of New York, has succeeded in producing local anæsthesia by introducing the anæsthetic into the skin by means of electricity. In the *New York Medical Journal*, of November 6th, he gives the method as follows: I procured an implement resembling the well-known instrument of Baunscheidt, but provided with many more fine needles than the conventional instrument of the shops. As in the Baunscheidt arrangement, this instrument is so constructed that by releasing a spring it is possible to thrust all the needles (about one hundred and fifty in number) into or through the integument, and, what is of paramount importance, *such perforation* is accomplished absolutely without pain. He first exsanguinates the part to be anæsthetized with an Esmarch bandage; and now applies a tourniquet above the bandage, and the latter is then removed. It is clear, therefore, that the whole district situated below the tourniquet, and which includes the territory which it is desired shall be rendered anæsthetic, is bloodless. Now, by means of the implement above described, he perforates the skin thoroughly throughout the entire zone which he desires to render anæsthetic. *This is accomplished without the slightest pain*, as already intimated. Owing to the exsanguinated condition of the part, these minute openings remain open somewhat as in a dead person. An oblong sponge electrode saturated with a two-and-a-half per cent. solution cocoaine is now secured over the perforated portion of the integument by means of an elastic strap. This electrode is connected with the positive pole of a galvanic battery, while the cathode is placed opposite the same (on the other side of the limb) or over some indifferent point. If, now, the plates of the battery are immersed and the current is gradually increased until there is a slight but well-marked sensation of warmth, the anæsthetic begins to exert its influence at once, so that in the course of from two to four minutes there is produced a condition of anæsthesia which enables one to thrust needles into the part to considerable depths without provoking pain. It is evident that where such a condition of things prevails in the integument the structures lying beneath may be readily anæsthetized, if one so desires, to any extent, and *without pain*, by the use of the hypodermic syringe, or by thrusting the needles a second time, still deeper. He says, I have employed this method recently to allay the hyperæsthesia which is such a troublesome feature of spinal irritation. I fancy, too, that it might render good service in certain obstinate conditions of tic doloreux, and I shall certainly try it in this and similar painful affections at the earliest convenient occasion.

We were pleased to meet Dr. L. H. Jones, of Clarkston, in this city a few days since. He has recently recovered from a severe attack of typho-malarial fever.

Mr. F. A. Scribner, representing Wm. Wood & Co., New York, dropped in on The Journal a few days since. He will, in the next few weeks, canvass Georgia and Alabama for the publications of this well-known house.

WE have received from Dr. J. Mc. F. Gaston, of this city, three reprints with the following titles: Obscure Impediments of the Intestinal Canal; Surgical Relations of the Ileo-cæcal Region, and Surgery of the Ileo-cæcal Connections, with new process for intestinal communication. The first and last appeared in *Gaillard's Journal*, and the second was a contribution to the surgical section of the last meeting of the American Medical Association, being illustrated with two wood-cuts showing the process for restoration of communication in stenosis or other permanent impediment of the intestinal canal. These papers cover satisfactorily a considerable and important division of abdomnial surgery, and give the results of experiments on inferior animals, of clinical observations, and of operative measures, with autopsic examinations, so as to prove instructive to the general practitioner as well as to the surgeon.

METEOROLOGICAL SUMMARY FOR OCTOBER, 1886, STATION, ATLANTA, GA.

| | |
|---|---|
| Mean Temperature, | 62.0 |
| Highest Temperature, 14th | 84.0 |
| Lowest Temperature, 28th, | 34.0 |
| Monthly Range of Temperature, | 50.0 |
| Greatest Daily Range of Temperature, | 29.0 |
| Least Daily Range of Temperature, | 10.0 |
| Mean Daily Range of Temperature, | 21.0 |
| Mean Daily Relative Humidity, | 61.0 |
| Prevailing Direction of wind, (7 a. m., 3 and 11 p. m.) | E. |
| Total movement of wind, | 6,552 miles. |
| Highest Velocity of wind, Direction and Date, | 24 miles E., 11th. |
| Total Precipitation. | .03 inches. |
| Number of Days on which .01 inch or more of Precipitation fell, | 2.0 |
| Number of Clear Days, | 19 |
| " " Fair " | 10 |
| " " Cloudy " | 2 |
| Dates of Frosts { Light, | None. |
| Dates of Frosts { Killing, | 28, 29, 30. |

## Figures and Tables

**Figure f1:**